# Temporal phylogeny and molecular characterization of echovirus 30 associated with aseptic meningitis outbreaks in China

**DOI:** 10.1186/s12985-021-01590-4

**Published:** 2021-06-06

**Authors:** Xiaoling Tian, Zhenzhi Han, Yulong He, Qiang Sun, Wenrui Wang, Wenbo Xu, Hongying Li, Yong Zhang

**Affiliations:** 1 Inner Mongolia Center for Disease Control and Prevention, Huhhot, 010031 People’s Republic of China; 2 WHO WPRO Regional Polio Reference Laboratory, National Health Commission Key Laboratory of biosafety, National Health Commission Key Laboratory of Medical Virology, National Institute for Viral Disease Control and Prevention, Chinese Center for Disease Control and Prevention, Beijing, 102206 People’s Republic of China; 3 Center for Biosafety Mega-Science, Chinese Academy of Sciences, Wuhan, 430071 People’s Republic of China; 4 Tongliao City Center for Disease Control and Prevention, Tongliao, 028000 People’s Republic of China; 5 Tongliao City Hospital, Tongliao, 028000 People’s Republic of China

**Keywords:** Echovirus 30 (E30), Molecular epidemiology, Phylodynamics, Aseptic meningitis

## Abstract

**Background:**

An outbreak of aseptic meningitis occurred from June to August 2016, in Inner Mongolia Autonomous Region, China.

**Methods:**

To determine its epidemiological characteristics, etiologic agent, and possible origin, specimens were collected for virus isolation and identification, followed by molecular epidemiological analysis.

**Results:**

A total of 363 patients were clinically diagnosed from June 1st to August 31st 2016, and most cases (63.1%, n = 229) were identified between June 22nd and July 17th, with children aged 6 to 12 years constituting the highest percentage (68.9%, *n* = 250). All viral isolates from this study belonged to genotype C of echovirus 30 (E30), which dominated transmission in China. To date, two E30 transmission lineages have been identified in China, of which Lineage 2 was predominant. We observed fluctuant progress of E30 genetic diversity, with Lineage 2 contributing to increased genetic diversity after 2002, whereas Lineage 1 was significant for the genetic diversity of E30 before 2002.

**Conclusions:**

We identified the epidemiological and etiological causes of an aseptic meningitis outbreak in Inner Mongolia in 2016, and found that Lineage 2 played an important role in recent outbreaks. Moreover, we found that Gansu province could play an important role in E30 spread and might be a possible origin site. Furthermore, Fujian, Shandong, Taiwan, and Zhejiang provinces also demonstrated significant involvement in E30 evolution and persistence over time in China.

**Supplementary Information:**

The online version contains supplementary material available at 10.1186/s12985-021-01590-4.

## Background

Aseptic meningitis (AM) is a clinical entity with inflammation of the brain parenchyma, it is an acute disease with many infectious and non-infectious causes, among which viral infection is the most common. A variety of viruses, such as Enteroviruses, mumps virus, adenovirus, and herpes simplex virus, can cause AM. Among these viruses, enteroviruses have become among the most common pathogens of viral meningitis in China [1]. The clinical manifestations of AM are mainly symptoms of meningeal inflammation, including fever, headache, and vomiting, as well as possible severe coma or convulsions. In some cases, enteroviruses cause large outbreaks with many severe and fatal cases [2, 3].

Echovirus 30 (E30) is a member of genus Enterovirus, family Picornaviridae, and belongs to human enterovirus species B (EV-B) together with 62 other serotypes [4]. Globally, E30 is among the most frequently identified enteroviruses and a major cause of aseptic meningitis (AM) [5–9]. In China, E30 outbreaks have been reported in many provinces, including Zhejiang, Jiangsu, Shandong, Henan, Fujian, and Guangdong [7, 9–13]. Further, E30 has a high isolation rate from patients with acute flaccid paralysis (AFP) and those with hand, foot, and mouth disease (HFMD), which is a new emerging concern that should promote extensive study on the inherent mechanisms involved [5–7, 12–14]. The AFP symptoms in immunosuppressed transplant recipients and outbreaks of acute myalgia and rhabdomyolysis in Brazil revealed severe non-neuropathic findings, expanding the variety of clinical symptoms caused by E30, which should be noticed more [15, 16].

In this study, we reported an AM outbreak caused by E30 in Tongliao City, Inner Mongolia, China, in 2016 and evaluated the epidemiological characteristics. We explored the genetic diversity and molecular characteristics of the isolates by sequence analysis of the *VP1* coding region and the full-length genome. Additionally, we investigated the phylodynamic diffusion patterns and recombination events of E30, which revealed the evolutionary features of E30 in China. This study offers insight into the outbreak characteristics and evolutionary dynamics of E30 in China.

## Methods

### Investigation of aseptic meningitis outbreaks and sample collection

**Fig. 1:**
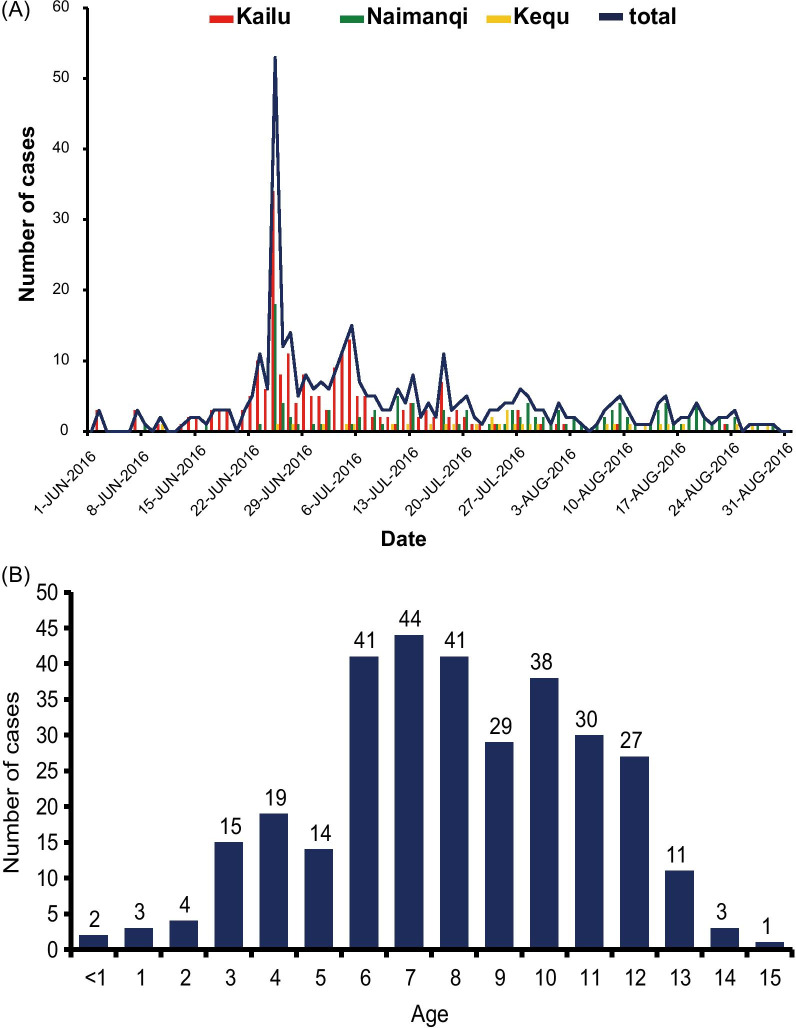
Epidemiology information of an outbreak of aseptic meningitis in Tongliao City, Inner Mongolia, China. **a** Cases of aseptic meningitis in townships of Tongliao city (Kailu, Naimanqi, and Kequ), Inner Mongolia, China, from June to August 2016. **b** Age distribution of cases reported during this outbreak

Based on the HFMD pathogen-surveillance system of the Inner Mongolia Provincial Center for Disease Control and Prevention, an epidemic of AM in Tongliao city from June to August 2016 was recorded, and representative samples were collected for pathogen detection. The local Center for Disease Control and Prevention (CDC) staff collected the clinical samples from several local hospitals having patients with viral meningitis. Use of their clinical samples was explained to the guardians of children, and written consent was provided by guardians of children to permit analysis of their clinical samples. Patients were classified as having a viral meningitis if they presented meningeal inflammation, such as fever, headache, and vomiting. In total, 363 cases were identified during this stage. Overall, 25 stool and cerebrospinal fluid (CSF) specimens were collected from children (age ≤ 15 years) at the peak and the end of the outbreak, which covered three counties of Inner Mongolia (Fig. 1a). The laboratory-confirmed evidence of AM comprised infection with EV-A71, CVA16, or other enteroviruses. Real-time reverse transcription polymerase chain reaction (RT-PCR) was the diagnostic method used for enterovirus detection, as described previously [17, 18].

### Virus isolation and molecular typing

For further etiological study, virus isolation from positive samples was performed in human rhabdomyosarcoma (RD) cells and human epithelium larynx (HEp-2) cells. Infected cell cultures were harvested after complete EV-like cytopathic effect (CPE) was observed. All experimental protocols were performed in accordance with the guidelines approved by the World Health Organization, as reported previously [19–21]. We ultimately obtained 12 isolates with complete EV-like CPE. Molecular typing of enterovirus isolates was performed by one-step RT-PCR amplification of the VP1 region using E30-specific primers described previously [13]. Unfortunately, we only harvested seven full-length VP1-coding-region sequences of E30 due to RT-PCR failure. The primers used for full-length genome sequencing of one E30 isolate were designed using a primer walking strategy.

### Bioinformatics analysis

Seven genomic sequences sampled from different patients, including two full-length genomes, were incorporated into the molecular epidemiological analysis. A phylogenetic tree for E30 genotyping was computed using the neighbor-joining method with 1000 bootstrap replicates in MEGA 7.0 software (https://www.megasoftware.net/), which implemented the same genotyping criteria described previously [9]. Genomic dataset mining for E30 was performed and included genomic sequences from GenBank and this study. To better represent E30 genetic diversity, we removed redundant E30 sequences, resulting in 354 E30 genomic sequences derived from various locations worldwide. Genome sequences were aligned using the MUSCLE method implemented in MEGA 7.0 software [22]. We constructed a maximum-likelihood phylogenetic tree using IQ-TREE software (http://www.iqtree.org/) with 1000 bootstrap replicates, and nucleotide acid-substitution models were inferred using ModelFinder with Bayesian information criteria [23, 24].

We implemented the Bayesian inference method in BEAST software (v.1.10.4) to investigate the phylodynamics of the genomes [25]. The maximum clade credibility (MCC) tree and coalescent-based Gaussian Markov random field (GMRF) skyride plots were explored using the SYM + G4 nucleotide-substitution model. We used the sampling times of genomic sequences to calibrate the molecular clock during each run. The time signals of datasets were assessed by the Bayesian evaluation of temporal signal (BETS) method and the root-to-tip method implemented in TempEst (v.1.5.3) [26, 27]. The results supported the sufficient temporal signals of the datasets in this study (See Additional file 1: Fig. S1). We implemented 15 dataset runs combined with one genome substitution model, three different clock models, and five different coalescent tree priors. All genome sequences for Bayesian inference and acquired from different provinces of China were coded as discrete states. The asymmetric substitution model was used to infer the asymmetrical transmission rates between any pairwise region state, including the Bayesian stochastic search variable selection option [28, 29]. Path sampling and stepping stone sampling analyses in BEAST were used to choose the best parameters of Bayesian phylogenetic models [30]. We checked the convergence and effective sample size (> 200) of the parameters using Tracer software (v.1.7) [31]. We summarized the MCC tree using TreeAnnotator software (v.1.10.4), with a burn-in of the first 10% of the sampled trees. The demographic dynamics of E30 in mainland of China were assessed using the GMRF method with a time-aware smoothing parameter [32]. The GMRF skyride plots were summarized and visualized using Tracer software (v.1.7.1), and ggtree (v.1.16.3) was used to manipulate the phylogenetic tree for the best performance [33, 34].

### Investigation of recombination signals

The Recombinant Detection Program (v.4.46; RDP4) was used to screen recombination signals in our entire set of genomic sequences using seven methods (RDP4, GENECONV, MaxChi, Bootscan, Chimaera, SiScan, and 3Seq) [35]. Briefly, the *P2* and *P3* coding-region sequences of the four strains were analyzed using the BLAST server (https://blast.ncbi.nlm.nih.gov/Blast.cgi) to compare their identity with sequences from GenBank. According to sequence similarities > 85%, these sequences were considered potential parents of the four strains and downloaded from GenBank. Phylogenetic incongruence between different regions with a p < 0.05 was considered strong evidence for recombination. We only considered recombination events that were identified by at least three methods. To confirm these putative recombination events, we utilized a smaller dataset that included the recombinant and parental strains for multiple screenings. The SimPlot program (v.3.5.1) was used for similarity plots and bootscanning analysis, with a 200-nucleotide window moving in 20-nucleotide steps [36].

## Results

### Profile of the AM outbreak in inner Mongolia, China

From June to August 2016, hospitalization for AM increased markedly at Tongliao People's Hospital in Inner Mongolia, China, which attracted the attention of the local CDC staff. An epidemiological curve of AM based on surveillance data from this time period in Tongliao city, Inner Mongolia, was thus generated (Fig. 1a). A total of 363 patients were clinically diagnosed from June 1st to August 31st 2016 and the epidemic spread over three townships of Tongliao city (Kailu, Naimanqi, and Kequ). Of the total number of cases, 63.1% (*n* = 229) were identified between June 22nd and July 17th, after which the cases gradually decreased, with certain turnovers during this progression. According to geographical distribution, Kailu and Naimanqi townships accounted for 58.4% (*n* = 212) and 32.8% (*n* = 119) of all cases, respectively, with children aged 0 to 5 years and 6 to 12 years constituting 15.7% (*n* = 57) and 68.9% (*n* = 250) of the cases, respectively (Fig. 1b). Children aged > 12 years accounted for only 4.1% (*n* = 15) of cases.

### Pathogen and genotype identification

**Fig. 2:**
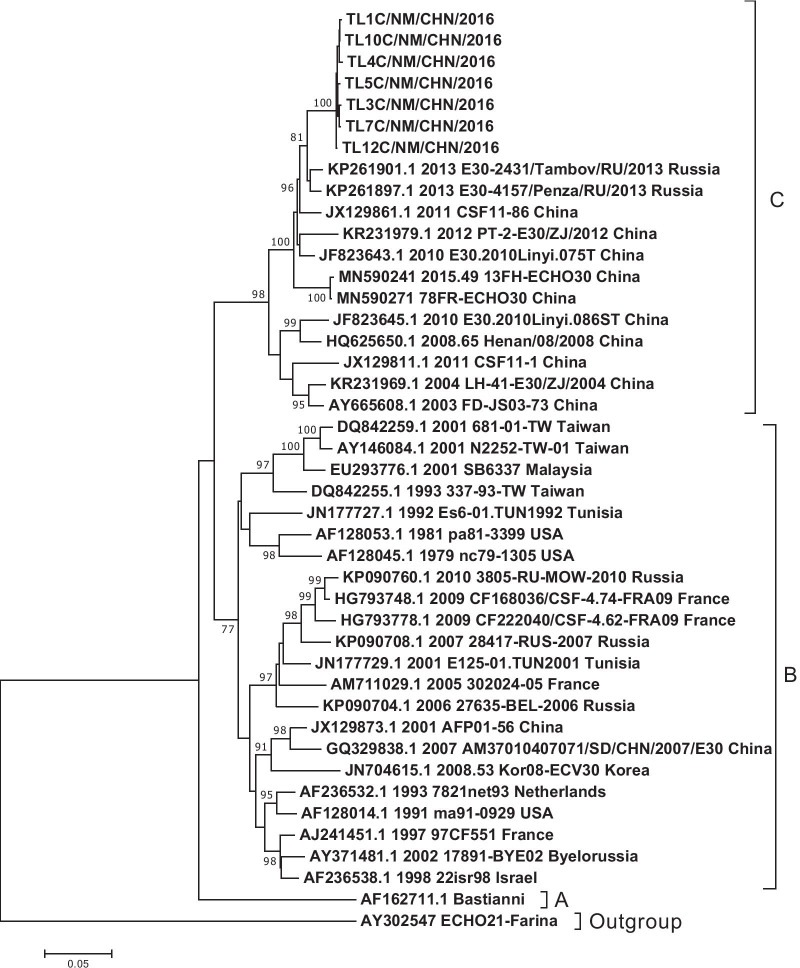
Generation of a phylogenetic tree. Tree based on the entire VP1 genome of 36 representative strains isolated from 1958 (Bastianni strain of the E30 prototype) to 2016, with the E-21 strain used as an outgroup and constructed using the neighbor-joining method with 1000 bootstrap replicates. Numbers at each node indicate the bootstrap support, and the letters (right) represent the E30 genotypes (A–C)

Twenty-five stool and CSF samples were inoculated into RD and HEp-2 cell lines for virus isolation, with complete EV-like CPE observed in 12 samples (48%). The full-length VP1 coding region of seven isolates was obtained and revealed ~ 98% nucleotide identity with E30 genomes from GenBank (accession no. KP985773). To confirm the serotype of the isolates, we constructed a neighbor-joining phylogenetic tree based on the EV-B prototypes (See Additional file 1: Fig. S1). The results showed that all isolates in this study clustered with the E30 prototype, confirming the BLAST results. The VP1 coding region of isolates in this study shared 99% nucleotide similarity between themselves, revealing a single origin of the collective outbreak. All identified enterovirus isolates met the strain-identification criteria with the homologous serotypes, including at least 75% nucleotide or 85% amino acid homology between the enterovirus isolates and the prototype enterovirus strain [37]. The genotype of the isolates in this study was investigated based on the genotyping criteria for E30 and previous reports [9, 38], with the results revealing that all isolates belonged to genotype C of E30 (Fig. 2). Moreover, genotype C showed 21.2% and 16.5% genetic divergence from genotypes A and B, respectively, with the mean genomic distance within genotypes B and C at 10.1% and 6.2%, respectively.

### Phylodynamics of E30 in China

Two transmission lineages of E30 had been previously identified in China according to a non-redundant genome dataset from China (See Additional file 2: Fig. S2). The maximum-likelihood tree based on this dataset showed that Lineage 1 circulated from 1988 to 2010 in China and tended to have recently disappeared. Strains of Lineage 2 were detected from 2003 to the present time and dominated the spread and occurrence of the collective outbreaks. The AM outbreak in this study was caused by Lineage 2 of E30, which showed a close phylogenetic relationship with the available strains isolated from Zhejiang, Jiangsu, Shandong, Sichuan, and Yunnan provinces of China (See Additional file 2: Fig. S2a).

**Fig. 3:**
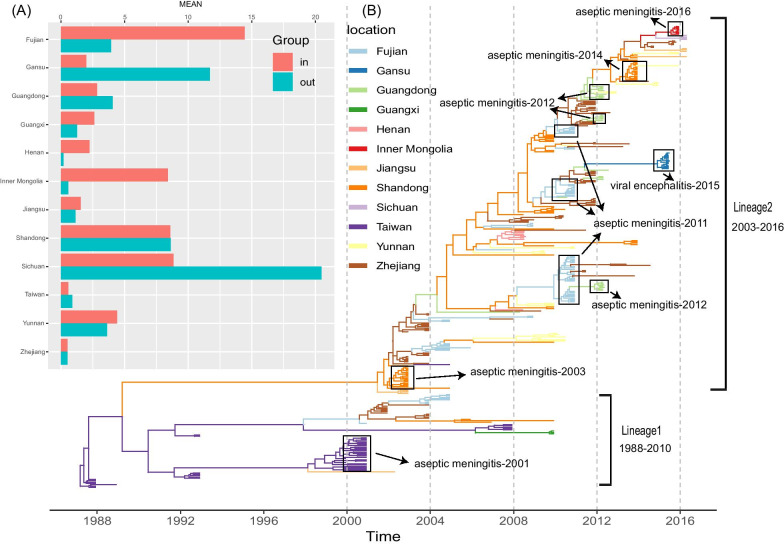
Temporal phylogenies and epidemic characteristics of E30. **a** Histogram of the average number of state counts based on different provinces. **b** The MCC phylogenetic tree based on the entire VP1 coding region in China and colored according to different provinces

We implemented the Bayesian method to infer the phylogenetic relationships and transmission tendencies of E30 in China, and the time signals of several datasets were checked using root-to-tip regression and BETS (See Additional file 3: Fig. S3). All results supported the existence of time signals in the datasets, with R2 values of 0.86 and high Bayes factors (BFs). The MCC tree showed the turnovers of E30 diffusion in China, revealing phylogenetic associations among different strains (Fig. 3). Consistent with the results of the maximum-likelihood tree, we observed two distant lineages in the MCC tree, which comprised a large number of genomes from outbreaks and surveillance reports [6, 11–13, 39]. The results showed that the branches of E30 isolated from different provinces aggregated together, and that E30 spread simultaneously in several provinces (Fig. 3b). For example, strains from Fujian province, China, were located in different clusters of Lineage 2, revealing the existence of multiple E30 variants in that outbreak. Moreover, we observed the evolution of E30 along a date clue, where strains from several provinces intersected, indicating the complicated diffusion dynamic of E30. Several AM-related outbreaks caused by E30 were recorded and annotated in the MCC tree, indicating that E30 frequently induced AM outbreaks in China following its evolution and transmission and revealing the active status of E30 in China.

**Fig. 4:**
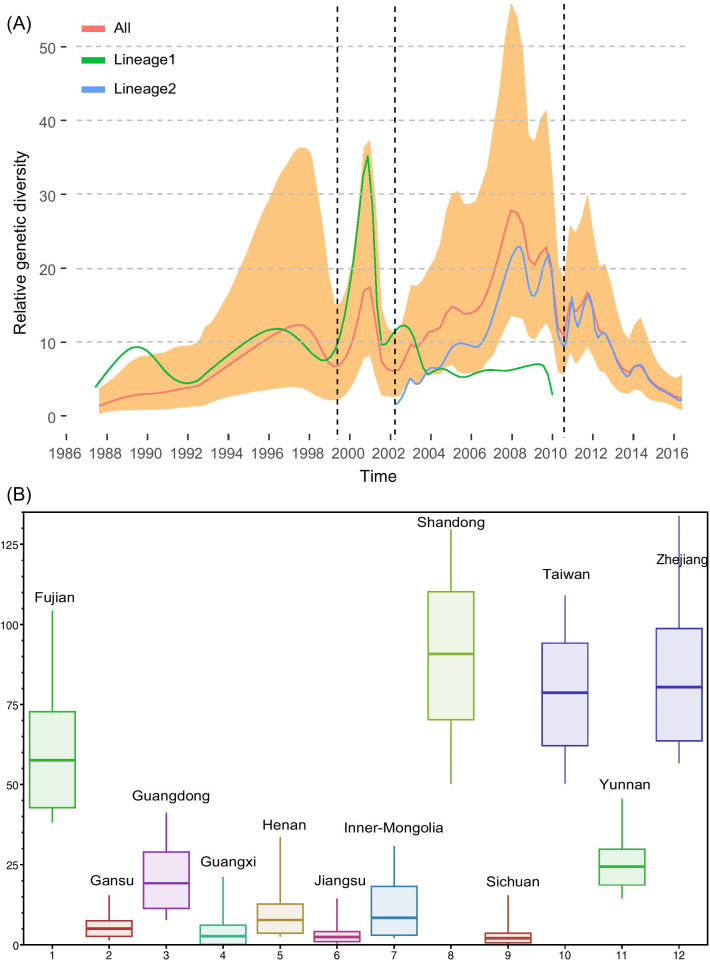
Relative genetic diversity of E30 genomes in China. **a** The X-axis represents the years, and the Y-axis shows the measure of genetic diversity (logarithmic scale of Neτ). Orange shading represents 95% credibility internally; and red, green, and blue lines show the median values of the E30 population size of three datasets (all data, Lineage1, and Lineage2), respectively. **b** Box plot of Markov reward values. The Y-axis shows the density distribution of the total time spent in a particular location

We observed that outward migration from Sichuan and Gansu provinces was dominant, whereas inward migration was dominant in Fujian, Henan, Guangxi, and Inner Mongolia (Fig. 3a). The outward-migration events of Sichuan and Gansu were observed through high BF and posterior probability (PP) values (PP = 0.5 and BF > 3) (See Additional file 7: Table S1). Further, the state counts were similar according to the outward and inward migrations in Shandong and Zhejiang, respectively. Other provinces of China showed more or less equal numbers of inward and outward migrations among different provinces. The Markov rewards values of Fujian, Shandong, Taiwan, and Zhejiang provinces were significantly higher than those of other provinces, indicating that these four provinces played a significant role in the evolution and persistence of E30 over time in China (Fig. 4b). Notably, Guangdong, Yunnan, Inner Mongolia, and Henan showed relatively higher Markov rewards values compared with those of Gansu, Guangxi, Jiangsu, and Sichuan provinces.

The genetic diversity of E30 showed a fluctuant progression during evolution following the date clue (Fig. 4a). We identified two top values of E30 genetic diversity during transmission when all genomes were used (Fig. 4a, red line). The genetic diversity peaked in 2001 and 2008, whereas it showed dynamic fluctuation between 2010 and 2014, and weaker genetic diversity was observed in the most recent outbreaks. Comparison of genetic diversity between the two lineages and all genomes indicated that the relative genetic diversity of Lineage 1 peaked near 2001 and dramatically reduced after 2002. The variable tendency of Lineage 2 showed similar characteristics relative that of all of the genomes (Fig. 4a, blue line). Lineage 2 contributed to the enhanced genetic diversity of E30 after 2002, whereas Lineage 1 played a significant role in the genetic diversity of E30 before 2002. The corresponding increased tendency between the two lineages and all genomes reflected the fluctuant switches of E30 genetic diversity.

### Investigation of recombination

To estimate the full-length genomic characteristics of E30, we obtained two full-length genomes. Similarity plots and bootscanning analyses revealed recombination events between the Inner Mongolia E30 isolate and the EV-B prototype at the 2A–2B junction region, implying the existence of potential recombination (See Additional file 4: Fig. S4). However, to locate the exact recombination activities between the two strains and the circulating enteroviruses, we scanned public databases to search for the recombination donor. Recombination analysis revealed overt evidence of inter-serotype recombination, as the recombination patterns of the two E30 strains in this study were similar. For the strain TL12C-NM-CHN-2016-E30 genome, breakpoint positions were identified at 3126–6627, 4066–4725, 4728–4920, and 4810–6621 (Table 1). Several EV-B strains, such as E-18, CV-B4, E-1, and CV-A9, were identified as minor putative parents using the RDP4 package. For the strain TL7C-NM-CHN-2016-E30 genome, we observed similar minor putative parents and breakpoint positions. We then constructed maximum-likelihood phylogenetic trees of the strains in this study based on the P1, P2, and P3 coding region along with the potential recombination donors, which confirmed the recombination events between the strains of this study and the E-18 strain LJ/0601/2019 (MN337405.1) (See Additional file 5: Fig. S5). Strain LJ/0601/2019 was isolated from the CSF of an adult with severe meningitis in 2019 in China. These results suggested that the role of recombination events in the evolutionary process of E30 needs to be assessed further.

**Table 1:** The results of recombination analysis inferred using RDP4 packages

Recombinant	Breakpoint position^ψ^		Region^δ^	Major parent	Minor parent	Methods*						
	Beginning breakpoint	Ending breakpoint				RDP	Geneconv	BootScan	MaxChi	Chimaera	SiScan	3Seq
TL12C-NM-CHN-2016-E-30-genome	3126(3070)	6627(6571)	P2, P3	KY888272.1_Echovirus_E-30_13-311	MN337405.1_Echovirus_E18_LJ/0601/2019	5.28 × 10^–119^	1.8 × 10^–112^	9.78 × 10^–100^	7.57 × 10^–27^	2.3 × 10^–9^	4.04 × 10^–87^	6.49 × 10^–13^
	4728(4672)	4920(4864)	P3	KU574629.1_Echovirus_E9_E-9/PMKA1322/THA/2011	JX308222.1_Human_coxsackievirus_B4_CVB4/GX/10	2.34 × 10^–9^	1.17 × 10^–4^	2.11 × 10^–8^	2.98 × 10^–4^	8.95 × 10^–3^	1.29 × 10^–4^	4.25 × 10^–7^
	4066(4010)	4725(4669)	P2, P3	MK815101.1_Echovirus_E-30_NO/538705/FAE/2016	JQ979292.1_Human_echovirus_1_polyprotein	1.24 × 10^–7^	8.9 × 10^–3^	4.58 × 10^–9^	1.11 × 10^–7^	1.09 × 10^–3^	5.25 × 10^–13^	NA
	4810(4754)	6621(6565)	P3	KY645964.1_Echovirus_E-30_16-I10	KY674976.1_Coxsackievirus_A9_16C8	7.42 × 10^–12^	5.25 × 10^–9^	1.37 × 10^–16^	5.54 × 10^–10^	3.47 × 10^–9^	8.19 × 10^–31^	7.13 × 10^–4^
TL7C-NM-CHN-2016-E-30-genome	3062(3006)	6629(6573)	P2, P3	KY888272.1_Echovirus_E-30_13-311	MN337405.1_Echovirus_E18_LJ/0601/2019	5.28 × 10^–119^	1.8 × 10^–112^	9.78 × 10^–100^	7.57 × 10^–27^	2.3 × 10^–9^	4.04 × 10^–87^	6.49 × 10^–13^
	4093(4037)	4725(4669)	P2, P3	MK815101.1_Echovirus_E-30_NO/538705/FAE/2016	JQ979292.1_Human_echovirus_1_polyprotein	1.24 × 10^–7^	8.9 × 10^–3^	4.58 × 10^–9^	1.11 × 10^–7^	1.09 × 10^–3^	5.25 × 10^–13^	NA
	4810(4754)	6621(6565)	P3	KY645964.1_Echovirus_E-30_16-I10	KY674976.1_Coxsackievirus_A9_16C8	7.42 × 10^–12^	5.25 × 10^–9^	1.37 × 10^–16^	5.54 × 10^–10^	3.47 × 10^–9^	8.19 × 10^–31^	7.13 × 10^–4^

^Ψ^Represents the breakpoint position in the alignment (the numbers within brackets represent the breakpoint position without gaps);

*Represents the P-value given by the RDP4 packages;

^δ^Represents the genomic structure of the enterovirus, which was related to recombination

## Discussion

Echoviruses are currently associated with a variety of human diseases, including asymptomatic infections, febrile illness, AM, and severe diseases in newborns. E30 is one of the main pathogens that cause AM, and acute myocarditis and exhibits strong infectivity [12, 38, 40]. E30 has shown wide circulation, including to the United States, Canada, France, Italy, Germany, England, Japan, South Korea, India, and China, and has demonstrated a highly epidemic trend in the previous 10 years [12, 40–44]. Therefore, understanding its pathogenesis and molecular epidemiology has important public health implications. During the period from June to August 2016, the number of hospitalized patients with AM increased sharply at Tongliao People’s Hospital, thereby surpassing the baseline of previous cases with AM in Tongliao city, Inner Mongolia.

We identified 363 patients during this time period in Tongliao city, Inner Mongolia, belonging to three townships that were affected by this outbreak, with Kailu and Naimanqi townships accounting for the greatest proportion of cases (*n* = 331, 91.2%). Compared with the homochromous numbers of AM cases in 2015, the number of AM patients increased significantly from June to August 2016 accompanied by a higher morbidity relative to other months. Children aged 6 to 12 years constituted the greatest proportion of all cases, consistent with previous reports [9, 39]. According to previous studies, viral meningitis outbreaks caused by E30 usually occurred from June to August in local regions and at the peak of the local enterovirus infection [6, 11, 13, 39, 44]. The epidemic then spread to other villages and towns, resulting in an outbreak within a short time period [6, 9, 13, 39, 45].

Outbreaks of aseptic meningitis caused by E30 occur mostly in densely populated eastern coastal areas, such as Jiangsu, Zhejiang, Shandong, Fujian, and Guangdong [6, 7, 9, 10, 12, 13, 39]. The outbreak in the present study was caused by Lineage 2, which showed a close phylogenetic relationship with the strains isolated from Zhejiang, Jiangsu, Shandong, Sichuan, and Yunnan provinces of China. Using the Bayesian method, we observed the turnover of E30 diffusion in China, implying its complex diffusion dynamic. The branches of E30 isolated from the different provinces aggregated together, and E30 spread simultaneously in several provinces simultaneously. E30 is widespread in China and has caused a large number of AM outbreaks. Moreover, we observed the evolution of E30 along the date clue, indicating gradual break outs in different provinces. The active activity status of E30 in China promoted its evolution and transmission, and the accumulation of genomic variants might play a significant role in local outbreaks of AM.

Mapping of transmission links through Bayesian inference showed that Sichuan and Gansu provided more outward migrations, whereas Fujian, Henan, Guangxi, and Inner Mongolia had more inward migrations. Additionally, more emigration events were found in Gansu, Sichuan, Guangxi, and Yunnan provinces, with high PP and BF support (See Additional file 7: Table S1). The outbreak in the present study possibly originated from these E30-migration events; however, we were unable to directly locate the accurate source of E30 due to low PP and BF support for Inner Mongolia (data not shown). However, the strains isolated from Sichuan province shared the closest phylogenetic relationships with those from Inner Mongolia (this study). Further, the high PP and BF support verified the transmission events from Gansu to Sichuan provinces, suggesting that the two outbreaks (Sichuan and Inner Mongolia) possibly possessed the same origin (See Additional file 6: Fig. S6, Additional file 7). Moreover, Gansu province could play an important role in E30 outbreaks and spread in Sichuan and Inner Mongolia based on the highest PP and BF support (See Additional file 7: Table S1). Furthermore, Gansu, Sichuan, and Inner Mongolia are neighbors in terms of their geographic distribution in China, suggesting local outbreaks and spread events among different provinces of China. We also observed that Fujian, Shandong, Taiwan, and Zhejiang provinces showed higher Markov rewards values as compared with other provinces, indicating that they played significant roles in E30 evolution and circulation over time in China. The eastern provinces of China were primary regions experiencing E30 infection and played an important role for further nationwide diffusion.

We then assessed the relative genetic diversity following the date clue, which showed fluctuant progression. Thus, the genetic diversity peaked ~ 2001 and ~ 2008, and different lineages showed polymorphic characteristics. The relative genetic diversity of Lineage 1 showed a similar fluctuant progression with that of all genomes before 2002, whereas that of Lineage 2 showed a similar fluctuant progression with that of all genomes after 2002. Therefore, Lineage 2 contributed to the enhanced genetic diversity of E30 after 2002, whereas Lineage 1 was important for the genetic diversity of E30 before 2002. This explains the contribution of fluctuant genetic diversity through the switch between different lineages.

The nucleotide and amino acid sequences in the *P2* and *P3* regions are highly conserved within an enterovirus species, and the *P2* and *P3* sequences do not correlate with EV serotypes due to frequent recombination; however, these sequences clearly distinguish different EV species [46]. In the present study, we identified overt evidence of inter-serotype recombination events. After screening several recombination signals, strain MN337405.1_Echovirus_E18_LJ/0601/2019 was detected as the putative recombination donor. This strain provided the raw recombination materials in the P2 and P3 coding regions for other recombinants. Frequent recombination and mutations in enteroviruses are recognized as the main mechanisms associated with their evolution, enabling their rapid response and adaption to new environments [46]. Accumulation of inter-species and intra-species recombination events is regarded as a strong driver for emergence and disappearance of certain enterovirus serotypes. Some studies have confirmed the ease of intra-species recombination events, and that EV-B is more susceptible to recombination [47–49]. The recombination events identified in the present study (i.e., the recombination donor was isolated from the CSF of an adult with severe meningitis in 2019 in China) verifies this. The recombination signals imply a vital role for E30 evolution and might be related to E30 pathogenicity and transmission.

## Conclusion

In this study, clinical specimens were collected from patients with AM, and the pathogens causing viral meningitis were screened. This process and subsequent investigation of the phylodynamic characteristics of the pathogens enhanced our understanding of the etiology of this outbreak of meningitis syndromes in Inner Mongolia. Investigation of the epidemiological and genetic characteristics of the E30 strains in Inner Mongolia provided a solid foundation for future detailed molecular epidemiological studies in China. Due to the recent increases in viral meningitis outbreaks in China, it is necessary to establish a pathogen-surveillance system in China that targets enterovirus-related syndromes in order to control enterovirus transmission and outbreaks [50, 51].

## Supplementary Information

**Additional file 1**. **Fig. S1**: The neighbour-joining phylogenetic tree, based on the *VP1* coding region, for serotyping. The EV-B prototype sequences of the *VP1* coding region and the genome sequences in this study were used.

**Additional file 2**. **Fig. S2**: (A). Magnification based on the red box in Figure S2 (B) and the branches coloured in red represent the E30 isolates from the collective outbreak in this study. (B). The midpoint-rooted maximum likelihood phylogenetic tree of E30 strains isolated from China. Scale bars represent the substitutions per site per year.

**Additional file 3**. **Fig. S3**: (A) Linear regression of root-to-tip divergence and sample dates. (B) Results of the Bayesian evaluation of temporal signal (BETS). The temporal signal of E-30 datasets was estimated using these two methods to assure sufficient temporal signals.

**Additional file 4**. **Fig. S4**: Similarity and bootscanning plot of EV-B prototypes and genomes isolated from this study, which was used to scan the recombination signals.

**Additional file 5**. **Fig. S5**: Maximum likelihood phylogenetic tree of E30 strains isolated from this study and the potential recombination donors. Scale bars represent the substitutions per site per year. (A), (B), and (C) represent the phylogenetic tree based on the P1, P2 and P3 coding regions.

**Additional file 6**. **Fig. S6**: Spatial transmission pathways of E30 inferred in this study using the Bayesian Stochastic Search Variable Selection (BSSVS) method. The solid black arrow shows the possible origin pathway of the outbreak in this study.

**Additional file 7**. **Table S1**: The migration events of E30 based on the *VP1* coding region.

## Data Availability

The data generated in this study are available at GenBank (accession nos. MW080371–MW080377) and China Virus Identification Net (accession nos. CVIN_AA002571-CVIN_AA002577).

## Abbreviations

AFP: Acute flaccid paralysis
AM: Aseptic meningitis
BETS: Bayesian evaluation of temporal signal
BF: Bayes factor
CDC: Center for Disease Control and Prevention
CPE: Cytopathic effect
CSF: Cerebrospinal fluid
E30: Echovirus 30
EV-B: Enterovirus species B
GMRF: Gaussian Markov random field
HEp-2: Human epithelium larynx cell line
HFMD: Hand, foot, and mouth disease
MCC: Maximum clade credibility
PP: Posterior probability
RD: Rhabdomyosarcoma cell line
RDP4: Recombinant Detection Program
RT-PCR: Reverse transcription polymerase chain reaction
