# Supplementary material for: Temporal phylogeny and molecular characterization of echovirus 30 associated with aseptic meningitis outbreaks in China
Source: Virol J. 2021 Jun 6;18:118. doi: 10.1186/s12985-021-01590-4 (PMC8182919; doi:10.1186/s12985-021-01590-4)
Supplement: Supplementary file 7 — Additional file 7. Table S1: The migration events of E30 based on the VP1 coding region. [file 12985_2021_1590_MOESM7_ESM.docx]

**Supplementary Information**

Temporal phylogeny and molecular characterization of echovirus 30 associated with aseptic meningitis outbreaks in China

Xiaoling Tian, Zhenzhi Han, Yulong He, Qiang Sun, Wenrui Wang, Wenbo Xu, Hongying Li, Yong Zhang

**Fig. S1. Neighbor-joining phylogenetic tree.** Tree based on the VP1 coding region for serotyping. The tree was generated using the EV-B prototype sequences of the VP1 coding region and the genome sequences in this study.

**Fig. S2.** **a**, Magnification of the red box shown in Figure 2 of the main text. Branches colored in red represent the E-30 isolates from the collective outbreak in this study. **b**, The midpoint-rooted maximum-likelihood phylogenetic tree of E-30 strains isolated from China. Scale bars represent substitutions per site per year.

**Fig. S3.** **a**, Linear regression of root-to-tip divergence and sample dates. **b**, BETS results. The temporal signal of E-30 datasets was estimated to ensure sufficient temporal signals.

**Fig. S4. Similarity and bootscanning plot of EV-B prototypes and genomes isolated during this study and used to scan the recombination signals.**

**Fig. S5. Maximum likelihood phylogenetic tree of E-30 strains isolated during this study and potential recombination donors.** **a–c**, The phylogenetic tree based on the P1, P2, and P3 coding regions, respectively. Scale bars represent the substitutions per site per year.

**Fig. S6. Spatial transmission pathways of E-30 inferred using Bayesian stochastic search variable selection.** The solid black arrow shows the possible origin pathway of the outbreak.

**Table S1. The migration events of E-30 based on the *VP1* coding region.**

| From | To | VP1 ^a^ | | |  |
| --- | --- | --- | --- | --- | --- |
|  |  | Mean migration rate | Indicator ^b^ | Bayes factor ^c^ | |
| Gansu | Yunnan | 1.006 | 0.66 | 20.42 | |
| Gansu | Sichuan | 0.949 | 1.00 | 18511.36 | |
| Yunnan | Guangxi | 0.939 | 0.90 | 92.72 | |
| Yunnan | Guangdong | 0.934 | 0.57 | 13.88 | |
| Jiangsu | Sichuan | 0.933 | 0.51 | 10.58 | |
| Guangxi | Guangdong | 0.964 | 1.00 | 4863.83 | |
| Guangxi | Sichuan | 0.965 | 0.95 | 189.30 | |
| Guangxi | Gansu | 0.953 | 0.97 | 298.41 | |
| Sichuan | Gansu | 0.949 | 0.88 | 78.08 | |
| Sichuan | Yunnan | 0.948 | 0.98 | 579.57 | |
| Fujian | Taiwan | 0.938 | 0.85 | 59.66 | |
| Guangxi | Jiangsu | 0.968 | 0.91 | 103.90 | |
| Guangxi | Shandong | 0.957 | 0.53 | 11.50 | |
| Sichuan | Fujian | 0.975 | 0.64 | 18.33 | |

^a^ E-30 coding region assessed for statistically supported migrations.

^b^ The posterior probability of an observed non-zero migration rate in the sampled trees.

^c^ Indicator values >0.5 and Bayes factor >3 of statistically supported migration rates.
